# Impact of rehabilitation dose on body mass index change in older acute patients with stroke: a retrospective observational study

**DOI:** 10.3389/fnut.2023.1270276

**Published:** 2023-12-05

**Authors:** Hiroyasu Murata, Syoichi Tashiro, Hayato Sakamoto, Rika Ishida, Mayuko Kuwabara, Kyohei Matsuda, Yoshiaki Shiokawa, Teruyuki Hirano, Ryo Momozaki, Keisuke Maeda, Hidetaka Wakabayashi, Shin Yamada

**Affiliations:** ^1^Department of Rehabilitation Medicine, Kyorin University Hospital, Mitaka, Japan; ^2^Department of Rehabilitation Medicine, Kyorin University School of Medicine, Mitaka, Japan; ^3^Department of Rehabilitation Medicine, Keio University School of Medicine, Tokyo, Japan; ^4^Department of Brain Surgery, Kyorin University, Mitaka, Japan; ^5^Department of Stroke and Cerebrovascular Medicine, Kyorin University, Mitaka, Japan; ^6^Department of Rehabilitation Medicine, Mie University Graduate School of Medicine, Tsu, Japan; ^7^Department of Geriatric Medicine, National Center for Geriatrics and Gerontology, Obu, Japan; ^8^Department of Rehabilitation Medicine, Tokyo Women’s Medical University Hospital, Shinjuku, Japan

**Keywords:** immobility, disuse, GNRI, malnutrition, stroke care unit, outcome, prognosis, elderly

## Abstract

**Background:**

It is established that a low body mass index (BMI) correlates with a diminished home discharge rate and a decline in activities of daily living (ADL) capacity among elderly stroke patients. Nevertheless, there exists a paucity of knowledge regarding strategies to mitigate BMI reduction during the acute phase. This investigation seeks to elucidate the impact of rehabilitation dose, as determined by both physical and occupational therapy, on BMI alterations, positing that a heightened rehabilitation dose could thwart BMI decline.

**Methods:**

This retrospective, observational study was conducted in the stroke unit of a university hospital. Enrollees comprised individuals aged ≥65 years, hospitalized for stroke, and subsequently relocated to rehabilitation facilities between January 2019 and November 2020. The percentage change in BMI (%ΔBMI) was calculated based on BMI values at admission and discharge. Multivariate multiple regression analysis was employed to ascertain the influence of rehabilitation dose on %ΔBMI.

**Results:**

A total of 187 patients were included in the analysis, of whom 94% experienced a reduction in BMI during acute hospitalization. Following adjustment for sociodemographic and clinical factors, multivariable analyzes revealed a positive association between rehabilitation dose and %ΔBMI (β = 0.338, *p* < 0.001).

**Conclusion:**

The findings of this study suggest that, in the context of acute stroke treatment, an augmented rehabilitation dose is associated with a diminished decrease in BMI.

## 1 Introduction

Nutritional status during the subacute phase of stroke is vital for the facilitation of intensive rehabilitation, the acquisition of muscle mass and fitness, functional recovery, and subsequent improved social outcomes. Research indicates that malnutrition at the time of transfer admission to subacute rehabilitation institutes from acute hospitals has a detrimental impact on rehabilitation outcomes (1–3). A low body mass index (BMI) at admission has adverse effects on social outcomes upon discharge from rehabilitation institutes (4) and the ability to perform activities of daily living (ADL) in stroke patients (5, 6). It is evident that usual dietary habits are associated with malnutrition (7) and sarcopenia (8). However, as nutrition cannot be improved prior to hospitalization, the focus should be on preventing malnutrition. Therefore, it is crucial to enhance nutritional status ahead of subacute stroke rehabilitation. Additionally, malnutrition and weight loss are frequently observed, with 34.6–55.1% of subacute stroke patients admitted to rehabilitation centers becoming malnourished after acute treatments (9). Patients often experience malnutrition and weight loss in the acute phase of stroke due to unconsciousness, impairments related to oral intake, comorbidities, and stress from the disease itself (9, 10). Overall, the prevention of malnutrition and low BMI, which are likely to occur during acute stroke treatment and care, is crucial for improving functional prognosis and social outcomes.

Older patients with stroke exhibit poorer functional prognoses and social outcomes compared to their younger counterparts (11, 12). Furthermore, nutritional decline is notably prevalent among older hospitalized patients (13) and is linked to compromised physical function, impaired rehabilitation outcomes (14), and an increased susceptibility to falls (15). Consequently, a multidisciplinary approach that includes nutrition is deemed essential (16). While researchers have identified factors contributing to short-term weight loss and malnutrition, such as feeding difficulties, low prealbumin levels, and ADL status (17), treatment interventions to address these issues remain challenging in practice due to their non-arbitrary modifiability. Therefore, it remains imperative to investigate the modifiable factors influencing malnutrition and weight loss in older patients.

Concurrently, early intensive rehabilitation intervention in the acute phase of stroke has been demonstrated to enhance physical function and abilities, including swallowing, motor function, and ADL (18, 19) and decrease hospital stay (20). However, Bernhardt et al. reported that patients with acute stroke tend to be physically inactive, spending a considerable amount of time in bed or at the bedside, even in the stroke unit (21). Researchers have found that bed rest is associated with a reduction in lean body mass (22) and muscle strength by 2–4% per day (23). Increasing the rehabilitation dose not only decreases bed rest time but may also prevent malnutrition and weight loss through mechanisms such as increased nutrient utilization (24) and reduced muscle catabolism in the general population (25). These findings suggest that a higher rehabilitation dose can positively influence malnutrition or weight loss in older patients with acute stroke. However, to our knowledge, no study has examined whether the rehabilitation dose, as the primary exercise opportunity for stroke patients undergoing acute treatment, affects weight loss and nutritional indices.

The study aimed to investigate the factors influencing BMI and nutritional indices at discharge, including rehabilitation dose, and to identify factors affecting nutritional status and preventing weight loss during the acute treatment and care of stroke.

## 2 Methods

### 2.1 Study design and subjects

**Figure 1:**
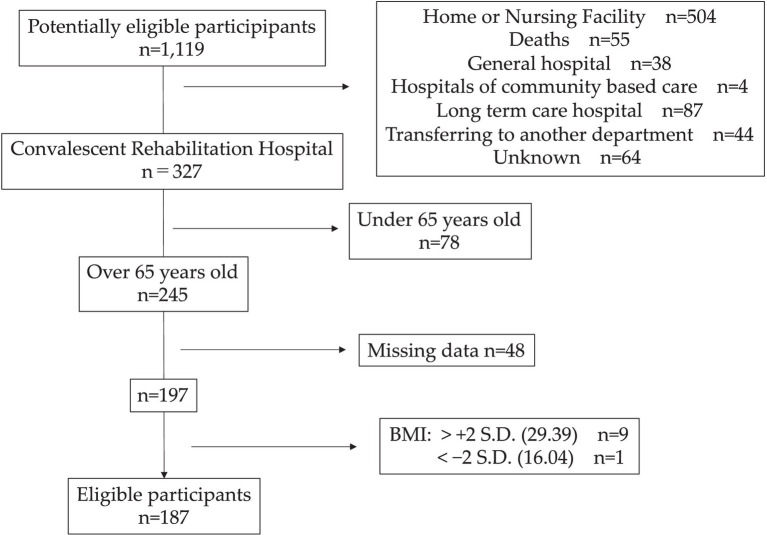
Flowchart of eligible participants screening and participation criteria.

We conducted a retrospective observational study involving 1,119 consecutive patients admitted to the stroke care unit at Kyorin University Hospital due to stroke between January 2019 and November 2020. This university hospital is a prominent regional institution equipped with a dedicated stroke care unit, excluding cases of subarachnoid hemorrhage, which are managed in the Department of Neurosurgery. The study encompassed patients who were (1) aged ≥65 years, (2) underwent rehabilitation, and (3) were transferred to rehabilitation centers for inpatient subacute stroke rehabilitation. Exclusion criteria included (1) severe comorbidities, encompassing osteoarticular disorders, metabolic diseases, neurodegenerative diseases, acute renal failure, and acute heart failure, which might impact rehabilitative treatments; (2) a BMI more than 2 standard deviations above or below the average, as aggressive individualized nutritional management might have been implemented; and (3) missing data (see Figure 1).

In this study, the acute and subacute phases of stroke were determined by the patient’s treatment status, not solely by the time elapsed since stroke onset. “Acute stroke” patients are those required acute medical treatment in the stroke care unit or ward, while “subacute stroke” patients are those undergoing rehabilitation in a dedicated rehabilitation hospital.

### 2.2 Sample size calculation

The sample size was calculated based on a previous study (26), considering a difference to be detected (0.8), variability of each group (1.6), power (0.9), and the proportion of the target group (0.5), resulting in a sample size of 172 cases.

### 2.3 Measurements

Various measurements were collected, including age, sex, height, weight, BMI, length of hospital stay, modified Rankin Scale (mRS) before stroke onset, type of stroke, level of consciousness at admission assessed with the Glasgow Coma Scale (GCS), National Institutes of Health Stroke Scale (NIHSS) scores at admission and discharge, serum albumin levels at admission and discharge, nutritional status assessed with the Geriatric Nutritional Risk Index (GNRI), and body energy expenditure and total energy expenditure calculated using the Harris-Benedict formula (27). Other data extracted from the medical records of each patient included energy intake, amount and content of rehabilitation, Functional Independence Measure (FIM) score at admission and discharge, and complications during hospitalization (pneumonia, urinary tract infection, and pressure ulcers).

The rate of change in BMI (%ΔBMI) was calculated using the following formula: (discharge BMI − admission BMI)/admission BMI × 100. In this study, we only used patients’ weights measured within 3 days of admission and 7 days of discharge. Patients were excluded if their weights were not measured within this period. When patients had difficulty standing, weight was measured on the bed or in a wheelchair, and the height was interviewed by the patient or his/her family.

The amount of food administered was confirmed by nurses, who checked the amount of food consumed and recorded it in electronic medical records daily. Total energy intake was recorded at three-time points after admission, having the day of admission as day 0: (1) days 1–3, (2) days 4–10, and (3) a week before discharge, excluding the day of discharge. Energy intake was defined as the average daily energy intake (kcal) divided by body weight for analyzes, regardless of the means of intake (oral, nasogastric tube, or intravenous infusion).

GNRI was calculated using the following formula: 14.89 × serum albumin level (g/dL) + [41.7 × (current weight/ideal weight)] (28). The rate of change in GNRI (%ΔGNRI) was calculated using the following formula: [(GNRI at discharge - GNRI at admission)/GNRI at admission × 100].

FIM scores were separately assessed for the motor domain (FIM-M) consisting of 13 sub-items and the cognitive domain (FIM-C) consisting of five sub-items. Tasks were scored on a 7-point ordinal scale ranging from full assistance to full independence (29). We used the Japanese version of FIM(TM) version 3.0 Data Management Service of the Uniform Data System for Medical Rehabilitation and the Center for Functional Assessment Research (30, 31) that has culturally relevant modifications for some of the items (32, 33).

The rehabilitation dose was defined as the total duration of physical and occupational therapy, excluding speech therapy. The total duration was normalized by the number of hospitalization days for analysis, classifying patients into high rehabilitation dose (HRD) and low rehabilitation dose (LRD) groups based on the median duration.

### 2.4 Outcome

The main outcome is %ΔBMI and the secondary outcome is %ΔGNRI.

### 2.5 Rehabilitation program

Patients were assessed by a board-certified rehabilitation physician, and individualized physical, occupational, and speech-language therapies were prescribed within 48 h, usually within 24 h. All rehabilitation programs were conducted on an individual basis. Rehabilitation aimed at improving functional impairments and ADL through various interventions such as range-of-motion training, muscle strengthening training, basic activity training (rolling, sitting, standing, and walking), swallowing training, and self-care training as soon as possible, according to the patient’s condition and the Japanese Stroke Treatment Guidelines (34). While the basic rehabilitation dose was determined by the rehabilitation physician’s instructions, adjustments were made by the patient’s physiotherapist and occupational therapist based on the patient’s condition and other factors on the day of rehabilitation.

### 2.6 Statistical analysis

Comparisons between the two groups were performed using the unpaired t-test, Mann–Whitney U test, and χ^2^ test, depending on the variables and their normality. The Shapiro–Wilk test was used to confirm normality. Spearman’s rank correlation was used for univariate analysis. The dependent variable was defined as %ΔBMI, and factors affecting %ΔBMI were selected based on previous studies and other data. Multiple regression analysis was performed using the forced entry method, omitting variables with a Spearman’s rank correlation *p* value greater than 0.1. Similarly, factors influencing %ΔGNRI were subjected to forced entry multiple regression analysis. In addition, further analysis was conducted excluding 54 patients who presented with infectious complications (pneumonia, urinary tract infection, and pressure ulceration) during hospitalization. The significance level was set at <5%. All statistical analyzes were performed using EZR (Saitama Medical Center, Jichi Medical University, Saitama, Japan) (35), which is a graphical user interface for R (The R Foundation for Statistical Computing, Vienna, Austria).

### 2.7 Ethical considerations

This study was approved by the Ethics Committee of the Kyorin University School of Medicine (Ethics No. R03-203). This study was conducted in accordance with the Declaration of Helsinki and the Ethical Guidelines for Medical and Health Research Involving Human Subjects.

## 3 Results

Participants were meticulously selected, as illustrated in Figure 1, resulting in the inclusion of 187 patients in the analysis. Patient characteristics are presented in Table 1. The median ΔBMI was −0.80 kg/m^2^, and the median %ΔBMI was −3.65%, with weight loss observed in 94% of patients. Upon stratification based on rehabilitation dose, the HRD group exhibited ΔBMI of −0.53 kg/m^2^ and %ΔBMI of −2.38%, while the LRD group demonstrated ΔBMI of −0.97 kg/m^2^ and %ΔBMI of −4.61%. Consequently, the HRD group experienced less weight loss compared to the LRD group (*p* < 0.05). No other parameters exhibited a significant difference, except for FIM-C scores at discharge, which were higher in the HRD group (*p* < 0.05).

**Table 1:** Comparison of high and low rehabilitation dose (HRD).

	Overall	LRD	HRD	*p*-value
Demographics				
Patients	187	93	94	
Age	78 (73–84)	79 (75–84)	77 (72–85)	0.160
Sex(man)	89	51	38	0.057
Height(cm)	156.8 (150–164)	158.0 (150.3–165.6)	156.0 (150.0–162.0)	0.109
Weight at admission(kg)	55.6 (48.1–63.0)	57.0 (49.4–64.0)	54.1 (47.0–61.9)	0.109
Weight at discharge(kg)	53.3 (46.0–60.1)	54.1 (46.8–61.1)	53.1 (45.6–59.8)	0.327
Length of hospital stay (days)	26 (22–34.5)	27 (22–37)	26 (21–33)	0.247
Pre-illness mRS	0 (0–2)	0 (0–2)	0 (0–2)	0.231
Clinical type				0.434
Cerebral infarction	127	66	61	
Cerebral hemorrhage	60	27	33	
GCS at admission	14 (13–15)	14 (13–15)	14 (14–15)	0.164
NIHSS at admission	7 (3.5–17)	7 (4–19)	7 (3–16)	0.559
NIHSS at discharge	3 (1–7)	3 (1–7)	3 (2–7)	0.613
Nutritional status				
BMI at admission(kg/m^2^)	22.32 (20.08–24.37)	22.52 (20.57–24.50)	22.01 (19.85–24.01)	0.381
BMI at discharge(kg/m^2^)	21.59 (19.51–23.23)	21.62 (19.54–23.27)	21.53 (19.45–23.06)	0.920
ΔBMI	−0.80 (−1.30− −0.38)	−0.97 (−1.41−−−0.64)	−0.53 (−1.2− −0.24)	<0.001
%ΔBMI	−3.65 (−6.00− −1.74)	−4.61 (−6.34 − −2.78)	−2.38 (−5.25− −0.95)	<0.001
ΔBMI ≧ 0	11 (6%)	2 (1%)	9 (5%)	
ΔBMI <0	176 (94%)	91 (49%)	85 (45%)	
Albumin at admission (g/dl)	3.9 (3.7–4.2)	3.9 (3.6–4.2)	3.9 (3.7–15.75)	0.938
Albumin at discharge (g/dl)	3.3 (3.0–3.7)	3.3 (3.0–3.6)	3.4 (3.1–3.8)	0.070
GNRI score at admission	100.55 (95.80–105.61)	100.76 (96.15–105.51)	100.08 (95.74–105.65)	0.955
GNRI score at discharge	91.08 (83.99–97.93)	88.77 (83.62–96.15)	91.93 (86.12–99.00)	0.125
GNRI change rate(%)	−8.86 (−16.01−−−2.07)	−11.23 (−16.38 — -3.85)	−8.18 (−14.48−−1.01)	0.156
BEE (kcal/day)	1107.31 (1019.55–1208.64)	1123.96 (1027.60–1233.60)	1094.32 (1019.42–1178.38)	0.137
TEE (kcal/day)	1353.02 (1240.94–1463.82)	1358.74 (1249.66–1479.13)	1335.48 (1239.15–1438.91)	0.332
Nutritional intake				
Day1〜3(kcal/day/kg)	20.29 (10.77–25.16)	20.01 (9.15–24.98)	21.38 (14.18–25.20)	0.198
Day4〜10(kcal/day/kg)	22.22 (18.06–25.45)	22.01 (19.03–25.17)	22.60 (18.04–25.53)	0.960
1 week to discharge(kcal/day/kg)	23.52 (19.47–27.07)	23.47 (19.38–27.42)	23.59 (19.68–26.87)	0.795
Rehabilitation and outcome				
Rehabilitation dose (min/day)	26.96 (23.49–33.21)	23.45 (21.08–25.00)	33.21 (28.84–37.33)	<0.001
ST intervention	164	83	81	0.657
FIM-M at admission	23.5 (13–35)	23.00 (13.00–35.00)	24.00 (13.00–35.00)	0.569
FIM-M at discharge	47 (24–63)	46.00 (20.50–62.00)	47.00 (25.00–65.00)	0.518
FIM-C at admission	16 (9–23.5)	14.00 (8.00–21.00)	18.00 (9.00–25.00)	0.052
FIM-C at discharge	23 (17–29.75)	21.00 (15.00–27.00)	25.50 (18.25–31.75)	0.004
FIM-total at admission	43 (22–58.5)	38.00 (22.00–56.00)	47.00 (23.00–60.75)	0.234
FIM-total at discharge	72 (46–92)	69.00 (39.00–88.50)	78.00 (48.00–95.00)	0.212
ΔFIM/ Length of hospital days	0.88 (0.41–1.43)	0.78 (0.32–1.36)	1.02 (0.50–1.47)	0.223
Complications				
Pneumonia	36	21	15	0.336
Urinary tract infections	18	10	8	0.786
Bedsore	6	3	2	1.000

LRD, Low rehabilitation dose; HRD, High rehabilitation dose; mRS, modified Rankin Scale; GCS, Glasgow Coma Scale; NIHSS, National Institutes of Health Stroke Scale; BMI, Body mass index; GNRI, Geriatric Nutritional Risk Index; BEE, Basal Energy Expenditure; TEE, Total Energy Expenditure; ST, Speech Therapy; FIM, Functional Independence Measure; FIM-M, Functional Independence Measure - Motor domain; FIM-C, Functional Independence Measure - Cognitive domain.

The results of Spearman’s rank correlation analysis are detailed in Table 2. %ΔBMI displayed a negative correlation with the admission NIHSS score (*r* = −0.149, *p* = 0.042) and length of hospital stay (*r* = −0.276, *p* < 0.001), while exhibiting positive correlations with the admission GCS (*r* = −0.203, *p* = 0.005), FIM-M (*r* = 0.157, *p* = 0.032), FIM-C (*r* = 0.182, *p* = 0.013), FIM-Total (*r* = 0.177, *p* = 0.015), energy intake on days 1–3 (*r* = 0.232, *p* = 0.001), energy intake on days 4–10 (*r* = 0.267, *p* < 0.001), energy intake the week before discharge (*r* = 0.156, *p* = 0.033), and rehabilitation dose (*r* = 0.368, *p* < 0.001). No significant associations were observed with age, admission BMI, or GNRI.

**Table 2:** Spearman’s rank coefficients with %BMI change.

	ρ	p-value
Age	0.066	0.369
BMI at admission(kg/m^2^)	−0.103	0.161
Length of hospital stay (days)	−0.276	<0.001
GCS at admission	0.203	0.005
NIHSS at admission	−0.149	0.042
GNRI score at admission	0.125	0.089
Rehabilitation dose	0.368	<0.001
FIM-M at admission	0.157	0.032
FIM-C at admission	0.182	0.013
FIM-total at admission	0.177	0.015
Nutritional intake day1-3(kcal/day/kg)	0.232	0.001
Nutritional intake day4-10(kcal/day/kg)	0.267	<0.001
Nutritional intake 1 week before discharge(kcal/day/kg)	0.156	0.033

BMI, Body mass index; GCS, Glasgow Coma Scale; NIHSS, National Institutes of Health Stroke Scale; GNRI, Geriatric Nutritional Risk Index; FIM, Functional Independence Measure; FIM-M, Functional Independence Measure - Motor domain; FIM-C, Functional Independence Measure - Cognitive domain.

To mitigate the potential negative impact of complications, further examinations were conducted by excluding cases that developed complications during hospitalization (pneumonia in 36 cases, urinary tract infection in 18 cases, and pressure ulcer in 6 cases). These results did not significantly differ from those of the analyzes including these patients (Supplementary Table S2).

The outcomes of multiple regression analysis with %ΔBMI as the dependent variable are summarized in Table 3. BMI at admission exhibited a negative effect (*β* = −0.300, *p* = 0.003), while the rehabilitation dose (*β* = 0.342, *p* < 0.001), mRS before admission (*β* = 0.146, *p* = 0.038), GNRI at admission (*β* = 0.387, *p* < 0.001), and energy intake on days 4–10 (*β* = 0.224, *p* = 0.018) demonstrated a positive effect on %ΔBMI.

**Table 3:** Multiple regression analysis with %BMI change as dependent variable.

	Unstandardized		Standardizedβ	*P*-value	95% CI	
	*B*	SE			Lower bound to upper bound	
Age	0.016	0.033	0.035	0.623	−0.049	0.082
BMI at admission(kg/m^2^)	−0.353	0.117	−0.300	0.003	−0.585	−0.121
mRS before admission	0.348	0.164	0.146	0.036	0.023	0.673
NIHSS at admission	0.021	0.035	0.054	0.554	−0.048	0.089
Rehabilitation dose	0.144	0.029	0.342	<0.001	0.086	0.201
FIM-M at admission	0.029	0.023	0.112	0.214	−0.016	0.075
FIM-C at admission	−0.028	0.037	−0.073	0.456	−0.101	0.046
GNRI at admission	0.164	0.039	0.387	<0.001	0.087	0.240
Nutritional intake day1-3(kcal/day/kg)	−0.005	0.037	−0.014	0.894	−0.079	0.069
Nutritional intake day4-10(kcal/day/kg)	0.111	0.047	0.224	0.018	0.019	0.203
Nutritional intake 1 week before discharge(kcal/day/kg)	−0.041	0.047	−0.076	0.381	−0.134	0.052

BMI, Body mass index; mRS, modified Rankin Scale; NIHSS, National Institutes of Health Stroke Scale; FIM-M, Functional Independence Measure - Motor domain; FIM-C, Functional Independence Measure - Cognitive domain; GNRI, Geriatric Nutritional Risk Index; BNP, Brain Natriuretic Peptide.

Multiple regression analysis, utilizing %ΔGNRI as the dependent variable, yielded results presented in Table 4. Energy intake in the week before discharge exhibited a negative effect (*β* = −0.157, *p* = 0.039), while BMI at admission (*β* = 0.294, *p* = 0.001) and GNRI at admission (*β* = 0.218, *p* = 0.007) demonstrated a positive effect on %ΔGNRI. Notably, the rehabilitation dose did not significantly affect %ΔGNRI.

**Table 4:** Multiple regression analysis with %GNRI change as dependent variable.

	Unstandardized		Standardizedβ	*P*-value	95% CI	
	*B*	SE			Lower bound to upper bound	
Age	−0.163	0.092	−0.110	0.079	−0.345	0.019
BMI at admission(kg/m^2^)	1.08	0.325	0.297	0.001	0.467	1.748
mRS before admission	0.500	0.454	0.066	0.272	−0.396	1.397
NIHSS at admission	−0.027	0.096	−0.022	0.781	−0.216	0.163
Rehabilitation dose	0.051	0.080	0.038	0.527	−0.107	0.208
FIM-M at admission	0.130	0.065	0.163	0.046	−0.002	0.257
FIM-C at admission	0.054	0.103	0.045	0.597	−0.148	0.257
GNRI at admission	0.293	0.107	0.219	0.007	0.081	0.505
Nutritional intake day1-3(kcal/day/kg)	0.064	0.103	0.057	0.538	−0.140	0.267
Nutritional intake day4-10(kcal/day/kg)	0.194	0.130	0.123	0.135	−0.061	0.449
Nutritional intake 1 week before discharge(kcal/day/kg)	−0.277	0.130	−0.161	0.034	−0.534	−0.021

BMI, Body mass index; mRS, modified Rankin Scale; NIHSS, National Institutes of Health Stroke Scale; FIM-M, Functional Independence Measure - Motor domain; FIM-C, Functional Independence Measure - Cognitive domain; GNRI, Geriatric Nutritional Risk Index.

## 4 Discussion

This study yielded two significant findings pertaining to weight loss in elderly patients with acute stroke undergoing transfer to rehabilitation institutes. Firstly, a substantial 94% of patients experienced weight loss, with a median reduction in BMI of −0.80 kg/m^2^, equivalent to −3.65% of the initial value. Secondly, the rehabilitation dose administered in acute stroke units exhibited a notable association with %ΔBMI. Notably, this study marks the first to highlight the pivotal role of the rehabilitation dose in averting nutritional deterioration during acute stroke treatment.

BMI reduction stands out as a straightforward indicator of malnutrition and proves valuable in routine nutritional management. Our findings align with prior studies reporting similar changes in BMI (−0.8 kg/m2) and weight loss (−2%) during acute stroke treatment (36). Additionally, the proportion of patients experiencing weight gain during acute stroke treatment was reported as 10.4% (37). While literature specific to the acute stroke unit has been scarce, studies focusing on rehabilitation outcomes in the subacute phase have indicated limitations imposed by malnutrition, as indicated by a decreased BMI (38, 39).

In this study, we have, for the first time, demonstrated a significant association between %ΔBMI and the rehabilitation dose within an acute stroke unit. Prior research has indicated that acute stroke patients often spend a considerable amount of time in bed (21, 40). Bed rest is linked to orthostatic hypotension, diminished cardiopulmonary function, and alterations in body composition (41–44), which may further impede patients’ activity out of the bed. Increased rehabilitation may have contributed to patients avoiding prolonged bed rest, potentially resulting in the preservation of muscle mass and subsequently mitigating weight loss. While previous studies have underscored the importance of muscle mass (45), measuring muscle mass was beyond the scope of this study and presents an avenue for future research.

Despite the patients in this study having a low NIHSS score post-acute treatment, two plausible reasons could account for this. Firstly, our focus was on patients necessitating subacute stroke rehabilitation. Secondly, the Japanese convalescent rehabilitation system initiates subacute rehabilitation relatively early. Given the impact of impairment on nutritional status, the low NIHSS cases in this study underscore the significance of intensifying the dose of acute stroke rehabilitation, particularly for those with severe impairment. The rehabilitation program employed in this study incorporates sitting and standing exercises, fostering early independence in activities of daily living (ADL). Consequently, the heightened level of activity outside the bed may play a crucial role in preventing weight loss. Additionally, exercise has been shown to enhance overall physiological function by increasing skeletal muscle glucose uptake (24) and promoting the release of myokines, which exert systemic and local anti-inflammatory effects (46). As a result, comprehensive rehabilitation measures contribute to averting weight loss in patients admitted to acute stroke care units.

In contrast to %ΔBMI, %ΔGNRI did not exhibit a significant correlation with the rehabilitation dose during the hospital stay. To the best of our knowledge, no study has undertaken a comparative analysis of the utility of BMI and GNRI in patients with acute stroke. GNRI, determined based on body weight, height, and serum albumin levels, reflects the protein provided to patients independently of the rehabilitation dose. The lack of significance in this aspect is not unexpected, considering the absence of nutritional intervention in the present study. Additionally, GNRI may not hold a superiority over BMI in this context as serum albumin levels are associated with inflammatory responses and electrolyte abnormalities (47), common occurrences during the acute phase of stroke (48).

Conversely, weight loss, directly linked to BMI, appears to have drawbacks. Previous reports have linked weight loss to complications such as respiratory infections, urinary tract infections, skin injuries, as well as stroke care-related factors including prolonged hospitalization and inadequate nutritional management (49–51). Nevertheless, further analysis excluding 54 patients who experienced infectious complications did not reveal statistical differences in the results for all patients. This suggests that the rehabilitation dose may positively impact the nutritional status of patients independently of complications. Consequently, the current findings propose that BMI remains a valuable general nutritional index in the acute phase of stroke.

Apart from the rehabilitation dose, multiple factors emerged as determinants of %ΔBMI and %ΔGNRI in the multiple regression analysis. High BMI and GNRI at admission, both exerting negative impacts, suggested that a substantial reduction in energy intake post-stroke onset induced a significant decrease in these parameters in patients, possibly attributable to overnutrition. Notably, energy intake on days 4–10 showed a positive association with %ΔBMI. Patients who initiated oral intake might face malnutrition due to appetite loss or dysphagia, while those dependent on nasogastric feeding or intravenous hyperalimentation might not experience malnutrition. When a patient capable of oral intake was unable to eat sufficient amount due to factors like appetite loss or dysphagia, energy intake decreased, leading to a reduction in %ΔBMI. This finding aligns with reports indicating that energy intake during the first week post-acute hospitalization influences discharge to home (52). These insights, along with previous studies (53), underscore the importance of choosing an appropriate method for nutritional intake in the early phase of stroke. In contrast, energy intake in the week before discharge exhibited a negative effect on %ΔGNRI. Given that GNRI is calculated using body weight and serum albumin levels, and a decrease in serum albumin level was observed in the present study, the nutritional status close to the time of the serum albumin examination might be reflected.

This study has several limitations that warrant consideration. Firstly, being a single-center study conducted at a stroke care unit in a Japanese university hospital, the generalizability of the findings to diverse populations or settings may be restricted. Secondly, as a retrospective, observational study, it did not explore differences in rehabilitation maneuvers and loads. Given the multifaceted influence of various clinical factors on rehabilitation dose, especially in acute stroke units, it would be essential to assess these factors from multiple perspectives. Notably, the hospital’s practice of requesting nurses to perform rehabilitation on public holidays, although not considered in the dose of rehabilitation, could potentially introduce systematic variability. Future prospective research with strict indications for rehabilitation in terms of dose and implementation is warranted to elucidate the relationship between rehabilitation and nutritional status. Thirdly, the study focused exclusively on patients requiring subacute inpatient rehabilitation after acute stroke treatment. While the availability of subacute rehabilitation was less influenced by economic status or social background due to the universal health insurance system in Japan, the decision to transfer to a rehabilitation hospital was influenced by the will of the patient, the patient’s family, and the medical team, introducing the possibility of selection bias (54). Lastly, the significance of weight change varies individually (55), especially in stroke patients, where categorization into overweight and underweight may be warranted. Particularly, more detailed interview regarding diet before the onset of stroke will also be informative.

## 5 Conclusion

In conclusion, this study unveils a significant association between rehabilitation dose and BMI change, which, in turn, correlates with the outcome of subacute phase rehabilitation. These findings underscore the importance of maximizing rehabilitation efforts in acute stroke care units.

## Data availability statement

The raw data supporting the conclusions of this article will be made available by the authors, without undue reservation.

## Ethics statement

The studies involving humans were approved by The Ethics Committee of the Kyorin University School of Medicine. The studies were conducted in accordance with the local legislation and institutional requirements. Written informed consent for participation was not required from the participants or the participants’ legal guardians/next of kin because This study was conducted using opt-out.

## Author contributions

HM: Conceptualization, Data curation, Formal analysis, Investigation, Methodology, Project administration, Writing – original draft, Writing – review & editing. ST: Conceptualization, Methodology, Supervision, Validation, Writing – original draft. HS: Investigation, Methodology, Writing – review & editing. RI: Investigation, Writing – review & editing. MK: Investigation, Writing – review & editing. KyM: Methodology, Writing – review & editing. YS: Supervision, Writing – review & editing. TH: Supervision, Writing – review & editing. RM: Conceptualization, Data curation, Methodology, Writing – review & editing. KeM: Conceptualization, Data curation, Methodology, Writing – review & editing. HW: Conceptualization, Data curation, Methodology, Writing – review & editing. SY: Methodology, Resources, Supervision, Writing – review & editing.
