# Supplementary material for: Impact of rehabilitation dose on body mass index change in older acute patients with stroke: a retrospective observational study
Source: Front Nutr. 2023 Dec 5;10:1270276. doi: 10.3389/fnut.2023.1270276 (PMC10728649; doi:10.3389/fnut.2023.1270276)
Supplement: Supplementary file 1 [file Table_1.docx]

Supplement Table1 Spearman’s rank coefficients among different factors

|  | Age | BMI at admission(kg/m^2^) | BMI change rate(kg/m^2^) | Length of hospital stay (days) | GCS at  admission | NIHSS at  admission | Rehabilitation dose | FIM-M at admission | FIM-C at admission | FIM-total at admission | GNRI at admission | Nutritional intake day1-3(kcal/day/kg) | Nutritional intake day4-10(kcal/day/kg) | Nutritional intake 1 week before discharge(kcal/day/kg) |
| --- | --- | --- | --- | --- | --- | --- | --- | --- | --- | --- | --- | --- | --- | --- |
| Age | 1 | -0.172* | 0.074 | -0.008 | -0.112 | 0.089 | -0.175* | -0.091 | -0.148* | -0.306** | -0.247** | 0.019 | -0.102 | -0.157* |
| BMI at admission(kg/m^2^) |  | 1 | -0.103 | -0.070 | 0.100 | -0.177* | -0.096 | 0.222** | 0.073 | 0.215** | 0.669** | 0.167* | 0.094 | 0.078 |
| BMI change rate(kg/m^2^) |  |  | 1 | -0.276** | 0.057 | 0.023 | 0.349** | 0.157* | 0.182* | -0.063 | -0.023 | 0.021 | 0.093 | 0.027 |
| Length of hospital stay (days) |  |  |  | 1 | -0.376** | 0.394** | -0.098 | -0.398** | -0.364** | -0.415** | -0.158* | -0.386** | -0.216** | -0.066 |
| GCS at admission |  |  |  |  | 1 | -0.677** | 0.185* | 0.557** | 0.623** | 0.505** | 0.223** | 0.609** | 0.387** | 0.204** |
| NIHSS at admission |  |  |  |  |  | 1 | -0.11 | -0.601** | -0.547** | -0.511** | -0.250** | -0.598** | -0.326** | -0.145* |
| Rehabilitation dose |  |  |  |  |  |  | 1 |  |  | 0.142 | 0.017 | 0.055 | 0.085 | 0.070 |
| FIM-M at admission |  |  |  |  |  |  |  | 1 | 0.695** | 0.938** | 0.265** | 0.582** | 0.288** | 0.067 |
| FIM-C at admission |  |  |  |  |  |  |  |  | 1 | 0.884** | 0.144* | 0.561** | 0.353** | 0.634** |
| FIM-total at admission |  |  |  |  |  |  |  |  |  | 1 | 0.314** | 0.516** | 0.463** | 0.205** |
| GNRI at admission |  |  |  |  |  |  |  |  |  |  | 1 | 0.191** | 0.199** | 0.097 |
| Nutritional intake day1-3(kcal/day/kg) |  |  |  |  |  |  |  |  |  |  |  | 1 | 0.559** | 0.251** |
| Nutritional intake day4-10(kcal/day/kg) |  |  |  |  |  |  |  |  |  |  |  |  | 1 | 0.518** |
| Nutritional intake 1 week to discharge(kcal/day/kg) |  |  |  |  |  |  |  |  |  |  |  |  |  | 1 |

BMI: Body mass Index, NIHSS: National Institutes of Health Stroke Scale, FIM-M: Functional Independence Measure - Motor domain, FIM-C: Functional Independence Measure - Cognitive domain, GNRI: Geriatric Nutritional Risk Index. *p<0.05, **p<0.01.
