# Supplementary material for: Impact of rehabilitation dose on body mass index change in older acute patients with stroke: a retrospective observational study
Source: Front Nutr. 2023 Dec 5;10:1270276. doi: 10.3389/fnut.2023.1270276 (PMC10728649; doi:10.3389/fnut.2023.1270276)
Supplement: Supplementary file 2 [file Table_2.docx]

Supplement Table2 Spearman’s rank coefficients with %BMI change excluding cases with complications

|  | ρ | P-value |
| --- | --- | --- |
| Age | 0.084 | 0.315 |
| BMI at admission | -0.152 | 0.084 |
| Length of hospital days | -0.339 | <0.001 |
| GCS at admission | 0.175 | 0.045 |
| NIHSS at admission | -0.156 | 0.075 |
| GNRI score at admission | 0.088 | 0.316 |
| Rehabilitation dose | 0.429 | <0.001 |
| FIM-M at admission | 0.066 | 0.450 |
| FIM-C at admission | 0.109 | 0.215 |
| FIM-total at admission | 0.091 | 0.304 |
| Nutritional intake day1-3 | 0.255 | 0.003 |
| Nutritional intake day4-10 | 0.277 | 0.001 |
| Nutritional intake 1 week before discharge | 0.152 | 0.083 |

BMI: Body mass index, GCS: Glasgow Coma Scale, NIHSS: National Institutes of Health Stroke Scale, GNRI: Geriatric Nutritional Risk Index, FIM-M: Functional Independence Measure - Motor domain, FIM-C: Functional Independence Measure - Cognitive domain, FIM: Functional Independence Measure
